# Cold-Stressed Soybean Sensitivity to Charcoal Rot

**DOI:** 10.3390/plants15030395

**Published:** 2026-01-28

**Authors:** Tomislav Duvnjak, Aleksandra Sudarić, Jasenka Ćosić, Karolina Vrandečić, Tamara Siber, Maja Matoša Kočar, Nina Cvenić

**Affiliations:** 1 Agricultural Institute Osijek, Juzno Predgradje 17, 31000 Osijek, Croatia; aleksandra.sudaric@poljinos.hr (A.S.); maja.matosa@poljinos.hr (M.M.K.); nina.cvenic@poljinos.hr (N.C.); 2 Faculty of Agrobiotechnical Sciences Osijek, Vladimira Preloga 1, 31000 Osijek, Croatia; jcosic@fazos.hr (J.Ć.); kvrandecic@fazos.hr (K.V.); tsiber@fazos.hr (T.S.)

**Keywords:** *Macrophomina phaseolina*, predisposition, genotype, effect size, Area Under the Lesion Progression Curve (AULPC), Cold Predisposition Index (CPI)

## Abstract

Charcoal rot, caused by *Macrophomina phaseolina*, is an increasingly important constraint in soybean, particularly under hot and dry conditions. While heat and drought are known to favor disease development, short early-season cold spells—common in temperate regions—may predispose soybean to subsequent infection, yet this interaction remains poorly quantified. It was evaluated whether transient chilling increases charcoal rot severity and whether cultivar-specific differences modulate this predisposition. Nine commercial cultivars spanning MG 00, 0, and 0–I were grown in a controlled walk-in chamber under either optimal conditions (control) or a three-day cold spell initiated at the first fully expanded trifoliate (20–23 days after sowing, DAS). Standardized cut-stem inoculation was performed at 26 DAS, and stem lesion length was recorded every 3–4 days across five assessments at 3, 7, 10, 14, and 21 DPI. Two-way ANOVA (treatment, genotype, treatment × genotype) with Tukey’s HSD tested effects. Cold stress significantly increased lesion lengths at all assessments, with the strongest divergence at the earliest measurement. Genotype and treatment × genotype were also significant, revealing differential responses among cultivars; notably, one line (G9) showed consistently small treatment-induced increases. These results indicate that brief early-season cold exposure can predispose soybean to more severe charcoal rot, with the magnitude dependent on genotype and timing. Incorporating cold-stress predisposition into screening and breeding should enhance resilience under increasing climate variability.

## 1. Introduction

Soybean [*Glycine max* (L.) Merrill] is a globally cultivated oilseed crop [1] and an important legume that serves as a major source of high-quality protein. Its popularity has grown due to its dual role in livestock feed and human nutrition [2]. Over the past decade, soybean production in Europe has increased by more than 87% [3], while in the European Union, it has increased by 116% [4]. Although cultivation is primarily concentrated in southern and eastern Europe, production has been expanding into new regions, underscoring its growing economic and agronomic importance [5]. In Croatia, between 2019 and 2023, the area under soybean cultivation ranged from 76,690 to 90,670 ha [6].

Among the most detrimental biotic factors affecting soybean production is *Macrophomina phaseolina* (Tassi) Goid (Goidanich, 1947) [7], a globally distributed necrotrophic soil- and seed-borne fungal pathogen. It is the causal agent of several diseases, including charcoal rot (also known as dry root rot or crown rot), stem rot, root rot, and seedling blight [8,9,10]. Beyond soybean, *M. phaseolina* infects more than 500 plant species [11,12] across over 100 families [13], including cereals and other legumes [14]. The pathogen colonizes the vascular tissues of roots, disrupting the transport of water and nutrients to aerial parts of the plant. Characteristic symptoms include yellowing and premature senescence of leaves that remain attached by petioles, sloughing of cortical tissues, and grey discoloration due to abundant microsclerotia, often resulting in premature plant death [15,16,17]. On soybean, symptoms typically manifest as stunted growth, chlorosis, early leaf senescence, and incomplete pod filling, depending on the developmental stage at infection [18,19]. The fungus survives in soil primarily as microsclerotia, which can remain viable for up to 15 years [20,21], making disease management particularly challenging [22]. Populations of *M. phaseolina* are most abundant in the upper soil layer (0–5 cm), followed by the 20–30 cm depth [23].

*M. phaseolina* has a wide geographic distribution and thrives under hot and dry conditions [24,25,26]. Under high temperatures (30–35 °C) and low soil moisture (<60%), it can cause severe yield losses in soybean, significantly impacting farmers’ income [27]. Yield losses in experimental plots have been reported to range between 18 and 30% [28], while complete (100%) losses have been recorded in groundnut cultivars infected at the pre-emergence stage [29]. In the United States, soybean yield losses exceeding 1.9–2.0 million tons were attributed to charcoal rot between 2003 and 2012 [30]. Despite substantial research efforts, effective management of *M. phaseolina* remains difficult. Understanding host–pathogen interactions is therefore essential for identifying genotypes with promising genetic resistance [31]. Several genotypes from maturity groups MG III to MG V have been identified as moderately resistant [32]. Maturity groups (MGs) are an agronomic classification that ranks soybean cultivars by their adaptation to day length and temperature, which together determine flowering time and time to physiological maturity. The scale progresses from very early (≈MG 000–00) to very late (≈MG VII–VIII), with cultivars adapted to higher latitudes belonging to earlier MGs and those suited to lower latitudes/longer seasons belonging to later MGs. In Europe, production largely spans MG 000–II, while this study focuses on MG 00, 0, and 0–I, which are most relevant for Central/Eastern European environments. Because MG influences phenology and the environmental window plants experience, it is directly relevant to potential disease escape/susceptibility and to the selected rationale for comparing genotypes across early MGs under cold-stress predisposition and subsequent infection conditions [5,19]. The use of resistant cultivars remains one of the most effective strategies for managing charcoal rot [30], as genetic resistance minimizes fungicide dependence, reduces yield losses, and supports sustainable crop production [17,33,34].

In Croatia, precise and systematic economic assessments of yield losses caused by this ubiquitous pathogen are lacking; however, available literature reports yield reductions over three years ranging from 5% to 30%, depending on the crop and environmental conditions [35].

Given the trend of increasingly warm and dry summers, particularly in eastern Croatia [36], where soybean is primarily grown, *M. phaseolina* is expected to have an escalating impact on yields. The pathogen’s extensive host range will likely further contribute to its persistence and spread.

Climate change is expected to alter pathogen dynamics and host susceptibility through multiple mechanisms, including shifts in temperature and precipitation patterns, pathogen evolution, and the breakdown of host resistance [37,38,39]. Climate projections indicate a higher frequency of extreme weather events, such as storms, droughts, and heatwaves [40], which are likely to intensify disease outbreaks and expand the cultivation range of susceptible hosts [41]. These environmental fluctuations can disrupt plant physiology, reduce inherent resistance, and enhance pathogen virulence and dispersal, ultimately leading to decreased crop productivity and increased plant mortality [42,43,44].

Soybean is a thermophilic crop with an optimal germination temperature between 20 °C and 30 °C. When exposed to low soil and air temperatures (below 10–12 °C during germination and below 15 °C during early growth), plants experience metabolic and structural disturbances collectively referred to as cold stress [45]. These conditions impair membrane integrity, enzyme activity, and nutrient uptake, resulting in slower growth and a reduced defense capacity [19]. Cold stress also increases the accumulation of reactive oxygen species (ROS) and reduces the synthesis of phenolic compounds, lignin, and pathogenesis-related proteins—key components of the plant’s defense response [46,47]. Furthermore, it suppresses defense-related enzymes such as phenylalanine ammonia-lyase (PAL), peroxidase, and β-1,3-glucanase [48], thereby increasing plant susceptibility to opportunistic fungal pathogens.

Cold stress thus acts as a predisposing factor that heightens soybean vulnerability to fungal infections, particularly during germination and early vegetative growth. By weakening cellular defenses, prolonging seedling emergence, and modifying the soil microenvironment, low temperatures create favorable conditions for opportunistic and soilborne pathogens. Although *M. phaseolina* typically develops under high temperatures and drought, soybean plants weakened by early cold stress may exhibit increased susceptibility later in the season when water stress occurs [49].

The aim of this study was to evaluate the sensitivity of several commercial soybean cultivars from different maturity groups (MG 00, 0, and 0–I) to *M. phaseolina* infection under controlled cold-stress and optimal growth conditions. Specifically, the study aimed to determine whether cultivar-specific differences in susceptibility exist and how individual cultivars respond to the combined effects of biotic and abiotic stress.

It is hypothesized that a brief early-season cold episode predisposes soybean to more severe *M. phaseolina* infection and that genotypes differ in both baseline severity under optimal conditions and the magnitude of cold-induced escalation. The objectives were to (i) quantify lesion development with and without prior cold stress, (ii) compare genotypic responses across MG 00, 0, 0–I, and (iii) summarize performance using ΔL, ΔL% and Area Under the Lesion Progression Curve (AULPC)-derived Cold Predisposition Index (CPI) metrics.

## 2. Results

### 2.1. Overall Effects of Treatment and Genotype

Analysis of variance showed that treatment (T), genotype (G), and their interaction (T × G) were significant sources of variation (*p* < 0.05) for lesion length across all five assessments (Table 1; Supplementary Method S1, Tables S2–S4). Based on 720 plants (3600 lesion measurements), this indicates that both cold stress and genetic background influenced disease severity and that genotypes responded differentially under cold-stress conditions.

In addition, partial η^2^ indicated a large treatment effect at the earliest assessment (M1: partial η^2^ ≈ 0.60–0.65) and small-to-medium effects thereafter (M2–M5: partial η^2^ ≈ 0.10–0.15); genotype and T × G also showed small-to-medium effects across assessments (see Supplementary Methods S1).

At the first assessment (M1), the between-treatment gap was very large (ΔL% ≈ 2029%) because C lesions were still near zero at this early stage; in absolute terms, the difference was on the order of centimeters (ΔL in cm reported in Table S2). Thereafter, the relative difference stabilized (≈46–51% across M2–M5).

Two-way ANOVA revealed significant main effects of treatment and genotype, as well as a significant T × G interaction, across all five assessments; F- and *p*-values for each measurement (M1–M5) are summarized in Table 1.

### 2.2. First Measurement (3 DPI)

**Figure 1:**
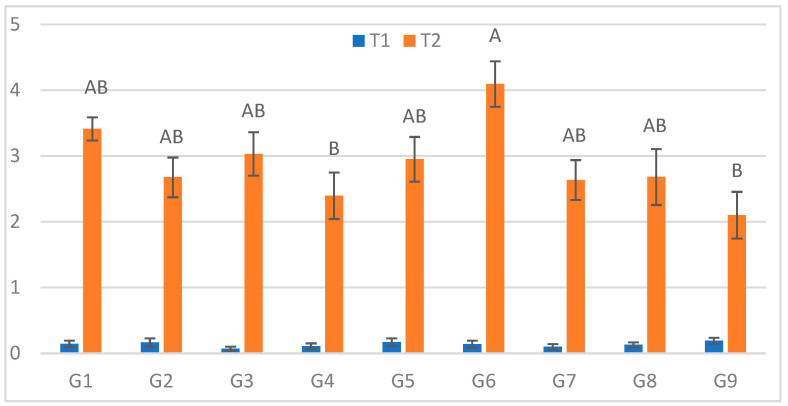
Infection lesion length (cm) at the first assessment by genotype (G1–G9) and treatment (C = optimal conditions; S = after a three-day cold spell (20–23 DAS)); *n* = 20 plants per genotype–treatment. Bars show means ± standard error (SE). Within-treatment lettering is shown with uppercase (C) and lowercase (S); means sharing a letter are not significantly different (Tukey’s HSD, α = 0.05). Letters are ordered from the highest to the lowest mean within each treatment.

At the first assessment (3 DPI; Figure 1), the longest mean lesion under cold stress (S) was recorded for G6 (4.10 cm), while the smallest lesions occurred on G9 (2.10 cm) and G4 (2.40 cm). The largest relative increase between treatments (ΔL%) was observed for G3 (4228.6%), and the smallest for G9 (1005.3%). The definitions and full calculations for the derived parameters [ΔL = L(S) − L(C); ΔL% = 100 × ΔL/L(C)] are given in Supplementary Methods S1, with genotype-wise values reported in Tables S2 and S3. Under optimal conditions (C), no significant genotypic differences were observed.

### 2.3. Second Measurement (7 DPI)

**Figure 2:**
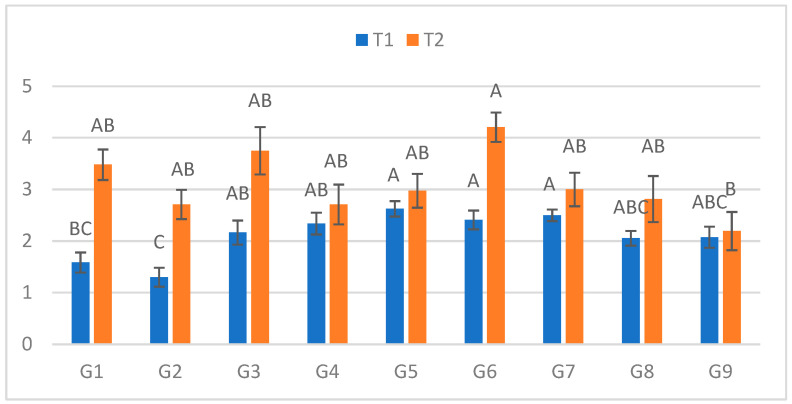
Infection lesion length (cm) at the second assessment by genotype (G1–G9) and treatment (C = optimal conditions; S = after three-day cold spell (20–23 DAS)); *n* = 20 plants per genotype–treatment. Bars show means ± standard error (SE). Within-treatment lettering is shown with uppercase (C) and lowercase (S); means sharing a letter are not significantly different (Tukey’s HSD, α = 0.05). Letters are ordered from the highest to the lowest mean within each treatment.

At the second assessment (Figure 2), significant genotypic differences emerged in both treatments. In C, lesions ranged from 1.30 cm (G2) to 2.625 cm (G5). In S, lesions ranged from 2.195 cm (G9) to 4.205 cm (G6). The greatest relative increase between treatments occurred for G1 (119.6%), while G9 again exhibited the smallest difference (5.8%).

### 2.4. Third Measurement (10 DPI)

**Figure 3:**
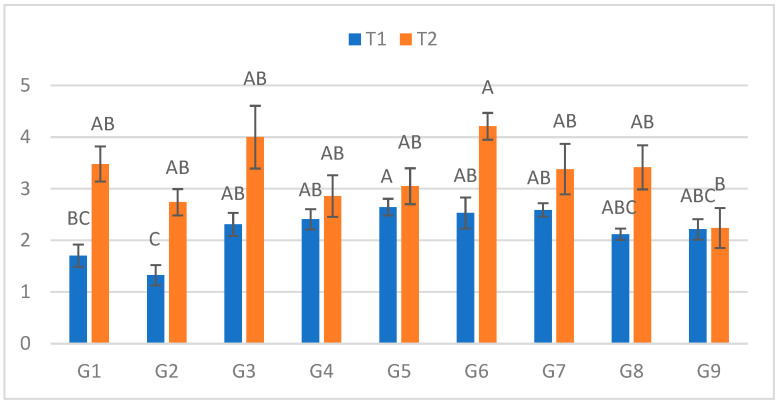
Infection lesion length (cm) at the third assessment (10 DPI) by genotype (G1–G9) and treatment (C = optimal conditions; S = after a three-day cold spell (20–23 DAS)); *n* = 20 plants per genotype–treatment. Bars show means ± standard error (SE). Within-treatment lettering is shown with uppercase (C) and lowercase (S); means sharing a letter are not significantly different (Tukey’s HSD, α = 0.05). Letters are ordered from the highest to the lowest mean within each treatment.

At the third measurement (Figure 3), the ranking of genotypes was consistent with previous assessments. In C, lesions ranged from 1.325 cm (G2) to 2.645 cm (G5), whereas in S, they ranged from 2.24 cm (G9) to 4.21 cm (G6). The largest treatment-induced increase was observed for G2 (106.8%), while G9 again showed minimal difference (1.1%).

### 2.5. Fourth Measurement (14 DPI)

**Figure 4:**
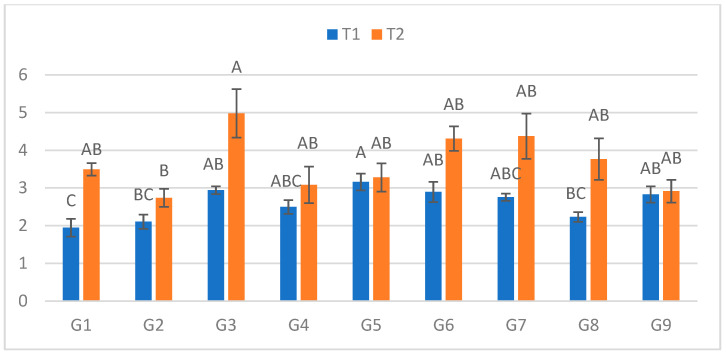
Infection lesion length (cm) at the fourth assessment by genotype (G1–G9) and treatment (C = optimal conditions; S = after a three-day cold spell (20–23 DAS)); *n* = 20 plants per genotype–treatment. Bars show means ± standard error (SE). Within-treatment lettering is shown with uppercase (C) and lowercase (S); means sharing a letter are not significantly different (Tukey’s HSD, α = 0.05). Letters are ordered from the highest to the lowest mean within each treatment.

At the fourth assessment (Figure 4), lesions in C ranged from 1.945 cm (G1) to 3.16 cm (G5). In S, lesions ranged from 2.74 cm (G2) to 4.98 cm (G3). The largest relative increase between treatments was detected in G1 (79.7%), whereas G9 once again showed the smallest increase (3%).

### 2.6. Fifth Measurement (21 DPI)

**Figure 5:**
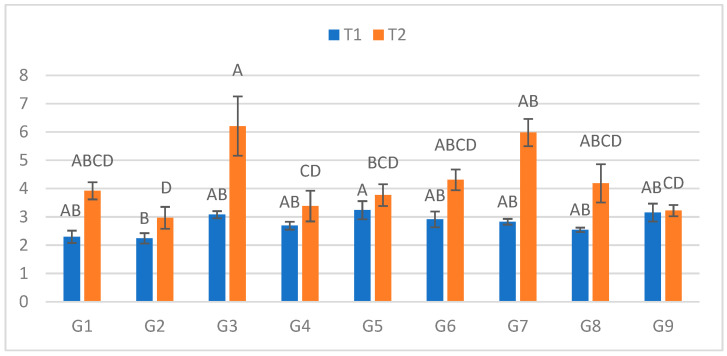
Infection lesion length (cm) at the fifth assessment by genotype (G1–G9) and treatment (C = optimal conditions; S = after a three-day cold spell (20–23 DAS)); *n* = 20 plants per genotype–treatment. Bars show means ± standard error (SE). Within-treatment lettering is shown with uppercase (C) and lowercase (S); means sharing a letter are not significantly different (Tukey’s HSD, α = 0.05). Letters are ordered from the highest to the lowest mean within each treatment.

At the final measurement (Figure 5), lesions in C ranged from 2.245 cm (G2) to 3.24 cm (G5). In S, lesion lengths ranged from 2.97 cm (G2) to 6.21 cm (G3). The greatest relative increase between treatments was observed in G7 (111.5%), and the smallest in G9 (2.2%).

## 3. Discussion

The present study demonstrated that transient cold stress significantly increased the severity of *M. phaseolina* infection in soybean and that the magnitude of this effect depended on both the timing of assessment and the genotype tested. Analysis of variance revealed that treatment, genotype, and their interaction were significant sources of variation in lesion length across all five measurements, confirming that both environmental and genetic factors, as well as their interaction, play a crucial role in the development of charcoal rot symptoms under controlled conditions.

Cold stress markedly enhanced lesion development, particularly at the earliest assessment, where the relative difference between treatments (C vs. S) was the greatest. This finding is consistent with the concept that low temperatures act as a predisposing factor by weakening host defenses and creating more favorable conditions for subsequent pathogen colonization. As described in previous studies, exposure to suboptimal temperatures disrupts membrane integrity, reduces enzyme activity, and impairs nutrient uptake [45], resulting in reduced overall physiological fitness of soybean seedlings and young plants [19].

At the biochemical level, cold stress is known to increase the accumulation of reactive oxygen species (ROS) and to reduce the synthesis of phenolic compounds, lignin, and pathogenesis-related proteins [46,47]. The suppression of key defense-related enzymes such as phenylalanine ammonia-lyase (PAL), peroxidase, and β-1,3-glucanase under cold conditions [48] further compromises the ability of plants to restrict pathogen ingress and spread. The results presented here, showing stronger infection and longer lesions in plants previously exposed to low temperatures, are in line with these physiological insights and support the hypothesis that short episodes of cold stress can substantially increase host susceptibility to necrotrophic fungal pathogens.

Although *M. phaseolina* is typically associated with high temperatures and drought [24,25,26,27], our findings indicate that prior exposure to cold can intensify disease development once favorable temperatures are re-established. This agrees with the notion that stress combinations or stress sequences—such as early cold stress followed by warmer, drier conditions—may exacerbate disease impact beyond what would be expected from individual stress factors alone [37,39]. In this context, cold stress appears not as an alternative to heat and drought stress, but as an additional abiotic factor that can predispose plants to more severe charcoal rot when typical conducive conditions for *M. phaseolina* occur later in the season [49].

The relative difference in lesion length between cold-stressed and control plants was highest at the first measurement and decreased in subsequent assessments, although absolute lesion lengths generally remained higher under cold stress. This temporal pattern suggests that the early phase of infection is particularly sensitive to prior physiological weakening induced by low temperatures. Once infection is established, lesion expansion appears to be driven more by inherent genotypic resistance or susceptibility and less by the initial cold stress event.

The stabilization of treatment differences over time is consistent with models of host–pathogen interaction in which abiotic stresses primarily influence the initial stages of colonization and penetration, whereas later stages reflect the balance between pathogen aggressiveness and host structural and biochemical defenses [19,44]. In practical terms, this implies that short cold spells during early vegetative stages can have disproportionate consequences for subsequent disease development, even if environmental conditions later in the season appear favorable for plant growth.

Genotype significantly affected lesion length in all measurements except the first assessment under optimal conditions, indicating that genetic variability in susceptibility becomes evident as infection progresses. Moreover, the significant T × G interaction across all time points confirms that genotypes responded differently to cold predisposition and that their relative performance was not constant across treatments.

Several consistent trends emerged. Genotype G9 repeatedly showed the smallest treatment-induced increase in lesion length across measurements, suggesting a relatively stable response to infection and potential partial tolerance to cold-stress-mediated predisposition. In contrast, genotypes such as G3, G6, and G7 often exhibited the greatest increases in lesions under cold stress, indicating heightened sensitivity when exposed to low temperatures prior to infection. These patterns agree with previous findings reporting genotypic variability in resistance to charcoal rot, in which some soybean lines exhibit reduced lesion lengths and lower yield losses in the presence of *M. phaseolina* [28,31,32].

The identification of genotypes with relatively stable performance under both optimal and cold-stress conditions is of particular interest for breeding programs. Resistant or less susceptible cultivars are recognized as one of the most effective and environmentally sustainable strategies for managing charcoal rot [30], as they reduce dependence on fungicides, minimize yield losses, and support long-term sustainable production [17,33,34]. The results suggest that genotypes similar to G9, which combine comparatively low lesion development and reduced sensitivity to cold stress, may represent promising candidates for further genetic and physiological characterization.

Climate change is expected to alter the epidemiology of plant diseases by modifying host physiology, pathogen biology, and the timing and frequency of abiotic stress events [36,37,38]. Projections indicate not only increased temperatures and more frequent droughts but also greater variability in early-season weather, including cold spells and fluctuating temperatures in temperate regions [40]. In such a scenario, soybean crops in regions like eastern Croatia may increasingly experience combinations of abiotic stresses that favor charcoal rot: early-season cold episodes followed by hot and dry periods later in the growing season.

The findings highlight that cold stress alone, even when transient, can significantly predispose soybean plants to more severe *M. phaseolina* infection. When combined with the known preference of this pathogen for high temperatures and low soil moisture [24,27,30], this suggests that climate variability may intensify charcoal rot risk in areas where soybean is expanding or where production already relies on marginal climatic conditions. In Croatia, where summers are becoming warmer and drier, and where no formal economic assessments of charcoal rot damage are currently available, the integration of cold-stress predisposition into risk models could improve the accuracy of future disease forecasts and management strategies.

This study operationalizes a climate-relevant cold-predisposition screen by combining a brief early-season chilling episode with a standardized cut-stem assay and by introducing rankable ΔL/ΔL% and CPI (AULPC-based) metrics that capture both baseline severity (C) and the magnitude of treatment-induced escalation (S vs. C). Together with morphological documentation and scale-barred controls, these components provide a reproducible framework for identifying genotypes with stable performance under climatic variability and for prioritizing entries in breeding pipelines.

### 3.1. Methodological Considerations and Limitations

The use of a controlled growth chamber and a single *M. phaseolina* isolate allowed a precise evaluation of treatment and genotype effects under standardized conditions. The cut-stem inoculation method applied at the V2 stage is widely used for resistance screening and offers high reproducibility and sensitivity [50]. However, several limitations should be considered when extrapolating these findings to field conditions.

First, only one isolate (MP1) was used, and it is well established that *M. phaseolina* populations can exhibit substantial genetic and pathogenic variability across regions and hosts [13,14]. Future studies should therefore include multiple isolates representing the diversity of field populations in Croatia and neighboring countries. Second, the cut-stem method bypasses some early infection stages occurring in natural soil or in residue-borne infections. Although this approach is highly informative for assessing relative lesion development and host responses, complementary experiments using soil inoculation under field or semi-controlled conditions would provide additional insight into root infection dynamics.

Third, the present study focused primarily on lesion length as a measure of disease severity. While this parameter is widely accepted and informative, integrating additional traits such as plant biomass, physiological status (e.g., photosynthetic activity), and yield components would help link lesion development more directly to agronomic performance. Finally, the cold-stress regime applied here was designed to simulate a realistic but specific scenario (three consecutive days of low temperature at the early vegetative stage). Other stress intensities, durations, and timings—especially in combination with drought—should be explored to better reflect the range of conditions expected under future climate scenarios.

### 3.2. Future Research and Breeding Perspectives

Building on the present screening, the next steps are threefold. First, field validation across locations with documented early-season cold spells should confirm genotype stability under natural weather variability and typical mid-season heat/drought sequences. Second, multi-isolate challenges (Croatian and regional diversity) will test whether the observed rankings persist across pathogen aggressiveness spectra. Third, integrating physiological and molecular markers (ROS metabolism, lignification, defense gene expression) with genomic selection frameworks could accelerate the identification of lines that combine low baseline severity with low CPI, thereby improving resilience to climate variability.

Two complementary breeding targets were distinguished: (i) baseline resistance under optimal conditions (low lesion development in C), and (ii) robustness to cold-stress predisposition (low ΔL, low ΔL%, and low CPI). The latter is best evaluated over time using AULPC and CPI, which condense lesion dynamics across assessments. This dataset, genotypes with comparatively low C lesion burden and low CPI (e.g., G9), represent stable candidates that maintain performance while limiting stress-induced escalation. Where agronomy prioritizes stress robustness (early cold spells expected), CPI-oriented ranking should be emphasized; where benign early seasons prevail, C performance may be weighted more strongly. For deployment decisions, a dual-criterion approach that reports both baseline severity (C) and cold-predisposition response (CPI) is therefore recommended, enabling environment-specific ranking.

Overall, this study demonstrates that even short episodes of cold stress can substantially increase susceptibility of soybean to *M. phaseolina* and that genotypes differ markedly in their responses to this combined abiotic–biotic stress scenario. These results underscore the importance of considering both climatic variability and host genetic resistance in the developing sustainable strategies for managing charcoal rot in soybean.

In practice, it is recommended to select lines that combine low C (good baseline) and low CPI (robust to cold predisposition); where early cold spells are frequent, CPI should be weighted more heavily, while in benign early seasons, C may be prioritized.

## 4. Material and Methods

### 4.1. Plant Material and Experimental Design

The experiment included nine soybean cultivars developed within the soybean breeding program of the Agricultural Institute Osijek (AIO, Osijek, Croatia). The entries represented three maturity groups (MGs): three MG 00, three MG 0, and three MG 0–I. For readability, genotypes are coded G1–G9 throughout, with MG mapping as follows: G1–G3, MG 00; G4–G6, MG 0; G7–G9, MG 0–I. Additional non-identifying metadata (origin category: breeding program vs. external supplier; status: registered cultivar vs. breeding line) are provided in Supplementary Table S4. To avoid brand-related bias and comply with research-use agreements, trade names are not shown.

In total, the experimental design comprised 9 genotypes × 2 treatments × 4 biological replicates × 10 plants (*n* = 720 plants), each measured at five time points (3600 lesion measurements). Two independent experiments were conducted under identical photoperiod conditions (16 h light/8 h dark) with a photosynthetic photon flux density (PPFD) of 300 μmol m^−2^ s^−1^. The only difference between treatments was the temperature regime, with plants in the stress treatment (S) exposed to a three-day cold-stress episode, whereas control plants (C) were maintained under optimal temperature conditions throughout. In both experimental time series, inoculation was performed at 26 days after sowing (DAS; V2 growth stage), as detailed in the Inoculation procedure.

Each experiment was arranged in a completely randomized design with four biological replicates per cultivar and ten plants per replicate. Plants were grown in plastic containers (600 × 400 × 200 mm) filled with 5.5 kg of soil (pH (CaCl_3_) = 5.7; N (NH_4_^+^ + NO_3_^−^) = 70 mg/L; P (P_2_O_5_) = 50 mg/L; K (K_2_O) = 90 mg/L; EC = 40 mS/m). Each tray contained six rows with alternating configurations of three and two planting spaces. Three genotypes were planted per tray, three trays were included per replicate, and twelve trays per treatment. The order of genotypes within each replicate was randomized, and trays were shuffled daily in the growth chamber before the lights were switched on. All plants were watered with tap water every second day.

### 4.2. Fungal Isolate and Inoculum Preparation

**Figure 6:**
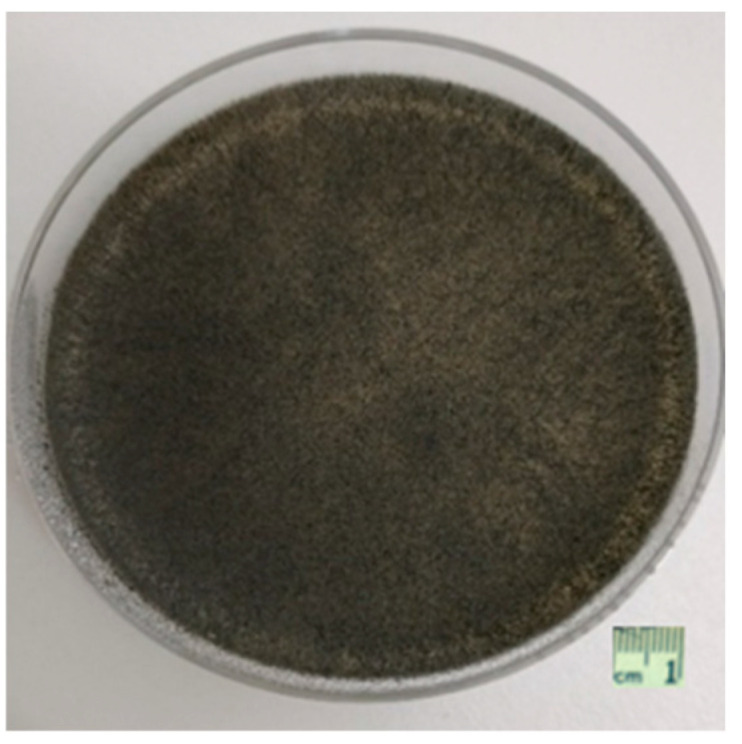
*M. phaseolina* isolate MP1 grown on potato dextrose agar (PDA) after 10 days of incubation at 28 ± 2 °C in the dark. The culture produced numerous dark, hard, ovoid microsclerotia used for inoculum preparation. Scale bar = 1 cm.

A ten-day-old culture of *M. phaseolina* isolate MP1 was used to prepare the inoculum. The MP1 isolate was obtained from a commercial hemp field in Vladislavci (45.4646950° N, 18.5674770° E), near Osijek, Croatia. To confirm morphological identification, a portion of the TEF1-α gene region was amplified using primers EF1-728F and EF2 [51,52], and the MP1 sequence (GenBank accession no. OQ389757; 212 bp) showed 100% nucleotide identity to the *M. phaseolina* reference sequence MG434668 [53]; phylogenetic placement is provided in Supplementary Figure S1. Culture plates were incubated at 28 ± 2 °C in the dark for 10 days, producing numerous dark, hard, ovoid microsclerotia (Figure 6).

### 4.3. Inoculation Procedure

**Figure 7:**
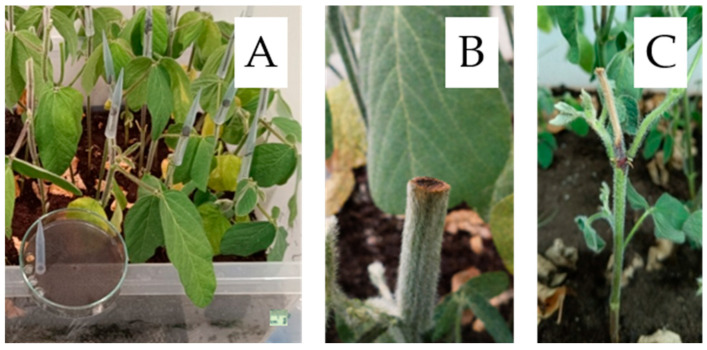
Cut-stem inoculation method used to screen soybean susceptibility to *Macrophomina phaseolina* at the V2 growth stage. (**A**) Overview of the experimental setup; scale bar = 1 cm. (**B**) Control plant after cutting, receiving a sterile PDA plug without mycelium. (**C**) Representative disease symptoms 21 days post-inoculation (DPI).

To assess cultivar susceptibility, ten plants per cultivar were inoculated at the V2 growth stage (fully developed second trifoliate leaf) using the cut-stem method [50], a standard approach for evaluating soybean resistance to *M. phaseolina*. In addition to inoculated plants, control plants received sterile PDA disks; representative images of both are provided with scale bars (Figure 6 and Figure 7). Plants were cut 25 mm above the unifoliolate node, and a circular agar disk containing fungal mycelium was placed onto the cut surface (Figure 7).

The open end of a 200 µL pipette tip was pressed into the margin of an actively growing *M. phaseolina* culture on potato dextrose agar (PDA), and an agar disk was cut and removed. The pipette tip containing the agar disk with fungal mycelium was immediately placed over the cut stem and gently pushed down to secure the agar disk in contact with the stem tissue. Control plants were treated with sterile PDA disks without mycelium.

Following inoculation, pipette tips were removed, and disease progression was visually assessed. Lesion length on stems was measured with a ruler every three to four days for a period of three weeks. Both time series were inoculated at 26 DAS. Agar plugs were standardized to a 200 µL pipette-tip bore and 10-day colony age, taken from the actively growing margin of replicate PDA plates from the same MP1 batch and randomized across plants. We did not quantify fungal biomass per plate; comparability was ensured by controlling colony age, growth zone, and plug size (Supplementary Methods S1).

### 4.4. Growth Conditions and Treatments

The experiment was conducted in a walk-in growth chamber (Fitoclima 10.000 HP; Aralab, Rio de Mouro, Portugal). Two treatments were applied in separate time series: (1) control (C), in which plants were grown under optimal conditions, and (2) cold stress (S), where plants were exposed to low temperatures for three consecutive days simulating early-spring cold spells. The stress treatment (S) was imposed at the V1 stage (first fully expanded trifoliolate), from 20 to 23 DAS; the control (C) remained under the optimal temperature regime. Growth-stage terminology follows [54].

The cold-stress treatment (S) began when soybean plants had developed the first fully expanded trifoliate leaf, approximately 20 days after sowing (DAS). The time required for full development of the first trifoliate was previously determined under identical conditions.

**Figure 8:**
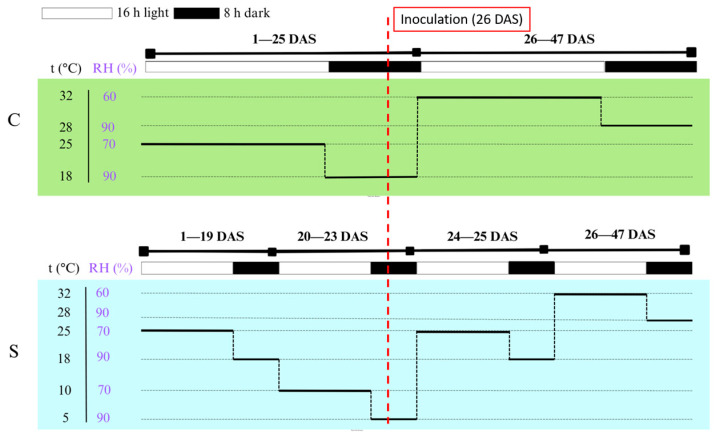
Growth-chamber settings for control (C) and cold-stress (S) treatments from day 1 to 47 after sowing (DAS). The diagram shows temperature (t °C), relative humidity (RH %), and light/dark cycles (16 h light/8 h dark; light intensity 300 µmol m^−2^ s^−1^, identical for C and S). During the cold-stress treatment, plants were exposed to low temperatures for three consecutive days (20–23 DAS) simulating early-spring cold spells. The vertical dashed line at 26 DAS marks the inoculation time point. Cold-stress window: 20–23 DAS (V1); inoculation: 26 DAS (V2).

The growth chamber maintained a 16 h/8 h (light/dark) photoperiod with a photosynthetic photon flux density (PPFD) of 300 μmol m^−2^ s^−1^, identical for both treatments. The only difference between treatments was the temperature regime during the three-day cold-stress episode; all other conditions, including light, were kept constant. Temperature and relative humidity profiles by phase (day/night; pre-/post-inoculation) are summarized in Figure 8. Environmental parameters were continuously monitored and logged using a FitoLog9000 data logger (Aralab, Rio de Mouro, Portugal). Inoculation was performed at 26 DAS (V2) (Figure 8, dashed line), after which lesion length was assessed at 3, 7, 10, 14, and 21 DPI.

### 4.5. Data Collection and Statistical Analysis (DPI Schedule)

Disease severity was assessed by measuring stem lesion length (mm) every 3 to 4 days over a 21-day period following inoculation. For each cultivar, ten plants per replicate were evaluated, and the mean lesion length per replicate was calculated. In addition to lesion length, visible disease symptoms (leaf chlorosis, wilting, premature senescence) were noted qualitatively to contextualize infection severity. These qualitative observations were not included in statistical analyses. Lesion length was assessed at five time points: 3, 7, 10, 14, and 21 days post-inoculation (DPI). We did not collect quantitative physiological covariates (e.g., SPAD chlorophyll, leaf water potential, soluble sugars); future trials will incorporate these metrics to mechanistically link cold predisposition, host physiology, and lesion dynamics.

The obtained data were subjected to statistical analysis to evaluate the effects of cultivar, treatment, and their interaction on *M. phaseolina* infection. Analysis of variance (ANOVA) was performed using the general linear model (GLM) procedure. We define treatment-induced increase as ΔL = L(S) − L(C) and the relative increase as ΔL% = 100 × ΔL/L(C); full details in Supplementary Methods S1. We also computed the area under the lesion progress curve (AULPC) and the Cold Predisposition Index (CPI = 100 × (AULPC_S − AULPC_C)/AULPC_C), with genotype-wise values reported in Supplementary Tables S2 and S3. When significant differences were detected (*p* < 0.05), means were separated using Tukey’s Honest Significant Difference (HSD) test. Normality of residuals and homogeneity of variances were verified prior to the ANOVA to ensure compliance with statistical assumptions. As a robustness check, we fit a linear mixed-effects model (random intercepts for replicate and plant, with repeated measures across time); inferences on Treatment, Genotype, and the Treatment × Genotype interaction matched the fixed-effects ANOVA (α = 0.05). Model details and outputs are provided in Supplementary Methods S1. For multiple comparisons, a compact letter display from Tukey’s HSD (α = 0.05) was used: means sharing a letter are not significantly different, whereas different letters indicate significant differences; letters are assigned from the highest to the lowest mean within each treatment (C and S analyzed separately).

All statistical analyses were performed using STATISTICA 13.0 (TIBCO Software Inc., Palo Alto, Santa Clara, CA, USA). Graphical representations of mean lesion lengths and standard errors were generated using GraphPad Prism 9.0 (GraphPad Software, San Diego, CA, USA).

## 5. Conclusions

This study provides clear evidence that transient cold stress significantly increases soybean susceptibility to *M. phaseolina*, particularly during the early stages of infection. The predisposition caused by cold stress was strongest at the initial measurement, indicating that early physiological disturbances play a key role in shaping subsequent disease development.

Genotypes differed markedly in their responses, with some lines (e.g., G9) showing relatively stable lesion development across treatments, while others exhibited substantial increases under cold stress. These results underscore the importance of incorporating cold-stress predisposition into resistance screening and highlight several genotypes as promising candidates for further evaluation and breeding for improved stability.

Given the expected rise in climatic variability—including early-season cold spells combined with mid-season heat and drought—understanding how abiotic stress interacts with charcoal rot will be crucial for developing resilient soybean cultivars. Integrating physiological studies, multi-isolate testing, and field validation will strengthen breeding strategies to mitigate the impacts of this increasingly important disease.

Practically, it is proposed to report two complementary criteria for cultivar choice: (1) baseline lesion severity under optimal conditions (C) and (2) Cold Predisposition Index (CPI) derived from AULPC. Lines that rank favorably on both axes (low C, low CPI) should be prioritized for advancement and deployment in regions prone to early cold spells.

## Figures and Tables

**Table 1:** Two-way ANOVA summary for stem lesion length across the five assessments (M1–M5). Factors are treatment (T), genotype (G), and their interaction (T × G). Values shown are F-statistics with corresponding *p*-values; common degrees of freedom are df(T) = 1, df(G) = 8, df(T × G) = 8, and residual df = 342.

Sources of Variation	Df	M1-F (*p*)	M2-F (*p*)	M3-F (*p*)	M4-F (*p*)	M5-F (*p*)
TREATMENT (T)	1	605.448 (<0.001)	52.973 (<0.001)	49.509 (<0.001)	44.541 (<0.001)	55.093 (<0.001)
GENOTYPE (G)	8	3.041 (0.003)	4.010 (<0.001)	3.529 (<0.001)	4.310 (<0.001)	5.042 (<0.001)
T × G	8	3.145 (0.002)	2.965 (0.003)	2.099 (0.035)	2.235 (0.025)	3.554 (<0.001)
Residuals	342					

Notes: M1–M5 denote the 1st–5th measurement time points. The model was fitted as a fixed-effects GLM with T and G; multiple comparisons in the text were conducted using Tukey’s HSD (*α* = 0.05). Very small *p*-values are reported as *p* < 0.001.

## Data Availability

Derived datasets (Tables S2 and S3) and analytical details (Methods S1) are provided in the Supplementary Materials; raw measurement data are available from the corresponding author on reasonable request.

## Supplementary Materials

The following supporting information can be downloaded at: https://www.mdpi.com/article/10.3390/plants15030395/s1, Figure S1: Phylogenetic identification of *Macrophomina phaseolina* isolate MP1 inferred from the TEF1-α gene region; Supplementary Method S1: Definitions and calculations of derived parameters and summary indices; Table S2: Derived lesion-length parameters by genotype and assessment time (DPI); Table S3: Time-course indices of lesion development by genotype; Table S4: Genotype metadata.

## References

[1] Šućur Elez J., Petrović K., Crnković M., Krsmanović S., Rajković M., Kaitović Ž., Malenčić Đ. (2023). Susceptibility of the Most Popular Soybean Cultivars in South-East Europe to *Macrophomina phaseolina* (Tassi) Goid. Plants.

[2] Galić Subašić D., Rapčan I., Jurišić M., Petrović D., Radočaj D. (2024). The Effect of Irrigation on the Yield and Soybean (*Glycine max* L. Merr.) Seed Germination in the Three Climatically Varying Years. Agriculture.

[3] FAOSTAT (2022). Crops and Livestock Products. FAO Statistics. https://www.fao.org/faostat/en/#search/Crops%20and%20livestock%20products%20%2B%20Total.

[4] Pospišil A., Pospišil M. (2024). Soybean Yield and Yield Components Depending on a Sowing Rate and Sowing Date. Agriculture.

[5] Karges K., Bellingrath-Kimura S.D., Watson C.A., Stoddard F.L., Halwani M., Reckling M. (2022). Agro-economic Prospects for Expanding Soybean Production Beyond its Current Northerly Limit in Europe. Eur. J. Agron..

[6] FAOSTAT (2025). FAO Crops Database. FAO Statistics. https://www.fao.org/faostat/en/#search/Industrial%20crops%252Fexport%20crops%20stat.

[7] Goidanich G. (1947). Revisione del genere *Macrophomina* Petrak. Ann. Sper. Agrar..

[8] Dhingra O.D., Sinclair J.B. (1978). Biology and Pathology of Macrophomina phaseolina.

[9] Ghosh T., Biswas M.K., Guin C., Roy P. (2018). A Review on Characterization, Therapeutic Approaches and Pathogenesis of *Macrophomina phaseolina*. Plant Cell Biotechnol. Mol. Biol..

[10] Reznikov S., Vellicce G.R., Mengistu A., Arias R.S., Gonzalez V., Lisi V.D., Ploper L.D. (2018). Disease Incidence of Charcoal Rot (*Macrophomina phaseolina*) on Soybean0 in North-Western Argentina and Genetic Characteristics of the Pathogen. Can. J. Plant Pathol..

[11] Wyllie T.D., Wyllie T.D., Scott D.H. (1988). Charcoal Rot of Soybeans—Current Status. Soybean Diseases of the North Central Region.

[12] Farr D.F., Rossman A.Y. (2015). Fungal Databases. Systematic Mycology and Microbiology Laboratory, ARS, USDA. http://nt.ars-grin.gov/fungaldatabases/.

[13] Marquez N., Giachero M.L., Declerck S., Ducasse D.A. (2021). *Macrophomina phaseolina*: General Characteristics of Pathogenicity and Methods of Control. Front. Plant Sci..

[14] Iqbal U., Mukhtar T. (2014). Morphological and Pathogenic Variability Among *Macrophomina phaseolina* Isolates Associated with Mungbean from Pakistan. Sci. World J..

[15] Short G.E., Wyllie T.D., Ammon V.D. (1978). Quantitative Enumeration of *Macrophomina phaseolina* in Soybean Tissues. Phytopathology.

[16] Sinclair J.B., Backman P.A. (1989). Compendium of Soybean Diseases.

[17] Smith G.S., Carvil O.N. (1997). Field Screening of Commercial and Experimental Soybean Cultivars for Their Reaction to *Macrophomina phaseolina*. Plant Dis..

[18] Gupta G.K., Sharma S.K., Ramteke R. (2012). Biology, Epidemiology and Management of *Macrophomina phaseolina* (Tassi) Goid with Special Reference to Charcoal Rot of Soybean. J. Phytopathol..

[19] Hartman G.L., Rupe J.C., Sikora E.J., Domier L.L., Davis J.A., Steffey K.L. (2015). Compendium of Soybean Diseases and Pests.

[20] Lodha S., Mawar R. (2020). Population Dynamics of *Macrophomina phaseolina* in Relation to Disease Management: A Review. J. Phytopathol..

[21] Purkayastha S., Kaur B., Dilbaghi N., Chaudhury A. (2006). Characterization of *Macrophomina phaseolina*, the Charcoal Rot Pathogen of Cluster Bean, Using Conventional Techniques and PCR-Based Molecular Markers. Plant Pathol..

[22] Short G.E., Wyllie T.D., Bristow P.R. (1980). Survival of *Macrophomina phaseolina* in Soil and in Residue of Soybean. Phytopathology.

[23] Lodha S., Mathur B.K., Solanki K.R. (1990). Factors Influencing Population Dynamics of *Macrophomina phaseolina* in Arid Soils. Plant Soil.

[24] Gray F.A., Kolp B.J., Mohamed M.A. (1990). A Disease Survey of Crops Grown in the Bay Region of Somalia, East Africa. FAO Plant Prod. Prot. Div. Bull..

[25] Diourte M., Starr J.L., Jeger M.J., Rosenow D.T., Stack J.P. (1995). Charcoal Rot Resistance and Effects of Water Stress on Disease Development in Sorghum. Plant Pathol..

[26] Wrather A., Shannon G., Balardin R., Carregal L., Escobar R., Gupta G.K., Tenuta A. (2010). Effect of Diseases on Soybean Yield in the Top Eight Producing Countries in 2006. Plant Health Prog..

[27] Kaur S., Dhillon G.S., Brar S.K., Vallad G.E., Chand R., Chauhan V.B. (2012). Biology, Economic Importance and Current Diagnostic Trends. Crit. Rev. Microbiol..

[28] Mengistu A., Smith J.R., Ray J.D., Bellaloui N. (2011). Seasonal Progress of Charcoal Rot and Its Impact on Soybean Productivity. Plant Dis..

[29] Sharma R.C., Bhowmik T.P. (1986). Estimation of Yield Losses in Groundnut Due to *Macrophomina phaseolina*. Indian J. Plant Pathol..

[30] Romero Luna M.P., Mueller D., Mengistu A., Singh A.K., Hartman G.L., Wise K. (2017). Advancing Our Understanding of Charcoal Rot in Soybeans. J. Integr. Pest Manag..

[31] Hemmati P., Zafari D., Mahmoodi S.B., Hashemi M., Gholamhoseini M., Dolatabadian A. (2018). Histopathology of Charcoal Rot Disease (*Macrophomina phaseolina*) in Resistant and Susceptible Cultivars of Soybean. Rhizosphere.

[32] Mengistu A., Arelli P.A., Bond J.P., Shannon G.J., Wrather A.J., Rupe J.B., Chen P., Little C.R., Canaday C.H., Newman M.A. (2011). Evaluation of Soybean Genotypes for Resistance to Charcoal Rot. Plant Health Prog..

[33] Bowen C., Schapaugh W. (1989). Relationships Among Charcoal Rot Infection, Yield, and Stability Estimates in Soybean Blends. Crop Sci..

[34] Bristow P., Wyllie T. (1984). Reaction of Soybean Cultivars to *Macrophomina phaseolina* as Seedlings in the Growth Chamber and Field. Trans. Mo. Acad. Sci..

[35] Croatian Meteorological and Hydrological Service (DHMZ) (2025). Climate Assessments—Temperature and Precipitation Anomalies for Central and Eastern Croatia. Climate Monitoring Reports. https://meteo.hr.

[36] Jurković D., Ćosić J., Vrandečić K. (2016). Pseudofungi and Fungi of Cereals and Arable Crops.

[37] Velasquez A.C., Castroverde C.D.M., He S.Y. (2018). Plant–Pathogen Warfare Under Changing Climate Conditions. Curr. Biol..

[38] Cohen S.P., Leach J.E. (2020). High Temperature–Induced Plant Disease Susceptibility: More Than the Sum of Its Parts. Curr. Opin. Plant Biol..

[39] Singh B.K., Delgado-Baquerizo M., Egidi E., Guirado E., Leach J.E., Liu H., Trivedi P. (2023). Climate Change Impacts on Plant Pathogens, Food Security and Paths Forward. Nat. Rev. Microbiol..

[40] Cook J., Oreskes N., Doran P.T., Anderegg W.R.L., Verheggen B., Maibach E.W., Carlton J., Lewandowsky S., Skuce A.G., GreenShow S.A. (2016). Consensus on Consensus: A Synthesis of Consensus Estimates on Human-Caused Global Warming. Environ. Res. Lett..

[41] Moullec F., Barrier N., Drira S., Guilhaumon F., Marsaleix P., Somot S., Ulses C., Velez L., Shin Y.J. (2019). An End-to-End Model Reveals Losers and Winners in a Warming Mediterranean Sea. Front. Mar. Sci..

[42] Ebi K.L., Ziska L.H., Yohe G.W. (2016). The Shape of Impacts to Come: Lessons and Opportunities for Adaptation from Uneven Increases in Global and Regional Temperatures. Clim. Change.

[43] Devendra C. (2012). Climate Change Threats and Effects: Challenges for Agriculture and Food Security.

[44] Lahlali R., Taoussi M., Laasli S.E., Gachara G., Ezzouggari R., Belabess Z., Aberkani K., Assouguem A., Meddich A., El Jarroudi M. (2024). Effects of Climate Change on Plant Pathogens and Host–Pathogen Interactions. Crop Environ..

[45] Allen L.H., Boote K.J., Reddy K., Hodges H. (2000). Crop Ecosystem Responses to Climate Change: Soybean. Climate Change and Global Crop Productivity.

[46] Hodges D.M., Lester G.E., Munro K.D., Toivonen P.M.A. (2004). Oxidative Stress: Importance for Postharvest Quality. HortScience.

[47] Prabhu S.A., Nemali K.S., Senthil-Kumar M. (2019). Abiotic Stress–Mediated Modulation of Defense Signaling Against Biotic Stress in Plants. Front. Plant Sci..

[48] Barka E.A., Nowak J., Clément C. (2006). Enhancement of Chilling Resistance of Inoculated Grapevine Plantlets with a Plant Growth–Promoting Rhizobacterium, *Burkholderia phytofirmans* Strain PsJN. Appl. Environ. Microbiol..

[49] Smith G.S., Wyllie T.D., Hartman G.L., Sinclair J.B., Rupe J.C. (1999). Charcoal Rot. Compendium of Soybean Diseases.

[50] Twizeyimana M., Hill C.B., Pawlowski M., Paul C., Hartman G.L. (2012). A Cut-Stem Inoculation Technique to Evaluate Soybean for Resistance to *Macrophomina phaseolina*. Plant Dis..

[51] Carbone I., Kohn L.M. (1999). A method for designing primer sets for speciation studies in filamentous ascomycetes. Mycologia.

[52] O’Donnell K., Kistler H.C., Cigelnik E., Ploetz R.C. (1998). Multiple evolutionary origins of the fungus causing Panama disease of banana: Concordant evidence from nuclear and mitochondrial gene genealogies. Proc. Natl. Acad. Sci. USA.

[53] Casano S.S., Hernández Cotan A., Marín Delgado A., F. García-Tejero I.F., Gómez Saavedra O., Aguado Puig A., de los Santos B. (2018). First Report of *Macrophomina phaseolina* on Hemp in Argentina. Plant Dis..

[54] Fehr W.R., Caviness C.E. (1977). Stages of Soybean Development. Special Report 80.

