# Supplementary material for: Cold-Stressed Soybean Sensitivity to Charcoal Rot
Source: Plants (Basel). 2026 Jan 28;15(3):395. doi: 10.3390/plants15030395 (PMC12899284; doi:10.3390/plants15030395)
Supplement: Supplementary file 1 [file plants-15-00395-s001.zip › plants-4050107-supplementary.pdf]

Supplementary Information

Manuscript: Cold-stressed soybean sensitivity to charcoal rot

S1. Supplementary Methods S1 — Definitions and calculations of derived parameters and summary indices

This Supplement describes the definitions and calculation procedures for the derived parameters used in the manuscript: the absolute treatment-induced increase (ΔL), the relative increase ($\Delta L\%$), and two time-course indices, the Area Under the Lesion Progress Curve (AULPC) and the Cold Predisposition Index (CPI). We also provide one worked example and indicate the accompanying summary tables (Tables S2–S3).

Notation

For each genotype g and assessment k, let $\bar{L}(g,k)^{(C)}$ denote the mean stem lesion length under control (T1) and $\bar{L}(g,k)^{(S)}$ under cold-stress (T2). Assessments were conducted at five days post-inoculation (DPI): $t = \{3, 7, 10, 14, 21\}$.

Definitions

(1) Absolute increase: $\Delta L(g,k) = \bar{L}(g,k)^{(S)} - \bar{L}(g,k)^{(C)}$ [cm]

(2) Relative increase: $\Delta L\%(g,k) = 100 \times (\bar{L}(g,k)^{(S)} - \bar{L}(g,k)^{(C)}) / \bar{L}(g,k)^{(C)}$ [%]

(3) AULPC (per treatment): $AULPC_g^{(T)} = \Sigma_{i=1}^{m-1} \frac{1}{2} \times [\bar{L}(g,t_i)^{(T)} + \bar{L}(g,t_{i+1})^{(T)}] \times (t_{i+1} - t_i), \quad T \in \{C,S\}$

(4) Cold Predisposition Index (CPI): $CPI_g = 100 \times (AULPC_g^{(S)} - AULPC_g^{(C)}) / AULPC_g^{(C)}$ [%]

Notes: Overbars indicate means across the 10 plants per genotype–treatment–replicate, then averaged across four biological replicates. For AULPC, the trapezoidal rule is used over DPI times t; units are cm·day. If $\bar{L}(g,k)^{(C)} = 0$ for any k, $\Delta L\%$ is undefined; in such rare cases, we report ΔL only and interpret CPI over the full time-course.

Worked example

Suppose for genotype Gx at 3 DPI we observe $\bar{L}(Gx,3)^{(C)} = 1.5$ cm and $\bar{L}(Gx,3)^{(S)} = 3.0$ cm. Then $\Delta L(Gx,3) = 1.5$ cm and $\Delta L\%(Gx,3) = 100 \times (3.0 - 1.5)/1.5 = 100\%$. If the control time-course at $t = \{3,7,10,14,21\}$ DPI is {1.5, 1.7, 1.9, 2.2, 2.5} and the stress time-course is {3.0, 3.3, 3.7, 4.0, 4.4}, then $AULPC_{Gx}^{(C)}$ and $AULPC_{Gx}^{(S)}$ are obtained by the trapezoidal rule and $CPI_{Gx} = 100 \times (AULPC_{Gx}^{(S)} - AULPC_{Gx}^{(C)}) / AULPC_{Gx}^{(C)}$.

Data handling and rounding

DPI values are fixed at 3, 7, 10, 14, and 21. Means are computed at the replicate level first, then averaged across replicates. When Tukey-adjusted comparisons are reported in the main text figures, bars indicate mean ± SE. For Supplementary Tables, ΔL is rounded to two decimals and $\Delta L\%$ to one decimal; AULPC values are shown to two decimals.

Supplementary Methods S1.1. Molecular identification of isolate MP1 (TEF1-α)

To confirm the morphological identification, a portion of the translation elongation factor 1-alpha (TEF1-α) gene region was amplified using primers EF1-728F and EF2. The resulting amplicon (212 bp; GenBank accession no. OQ389757) showed 100% nucleotide identity to *Macrophomina phaseolina* reference sequence MG434668 (GenBank). The phylogenetic placement of MP1 based on TEF1-α sequences is shown in Supplementary Figure S1. Although the fragment is short, it provided an unambiguous match to *M. phaseolina* in BLASTn and supported clustering with reference sequences.

Supplementary Figure S1. Phylogenetic identification of *Macrophomina phaseolina* isolate MP1 inferred from the TEF1-α gene region.

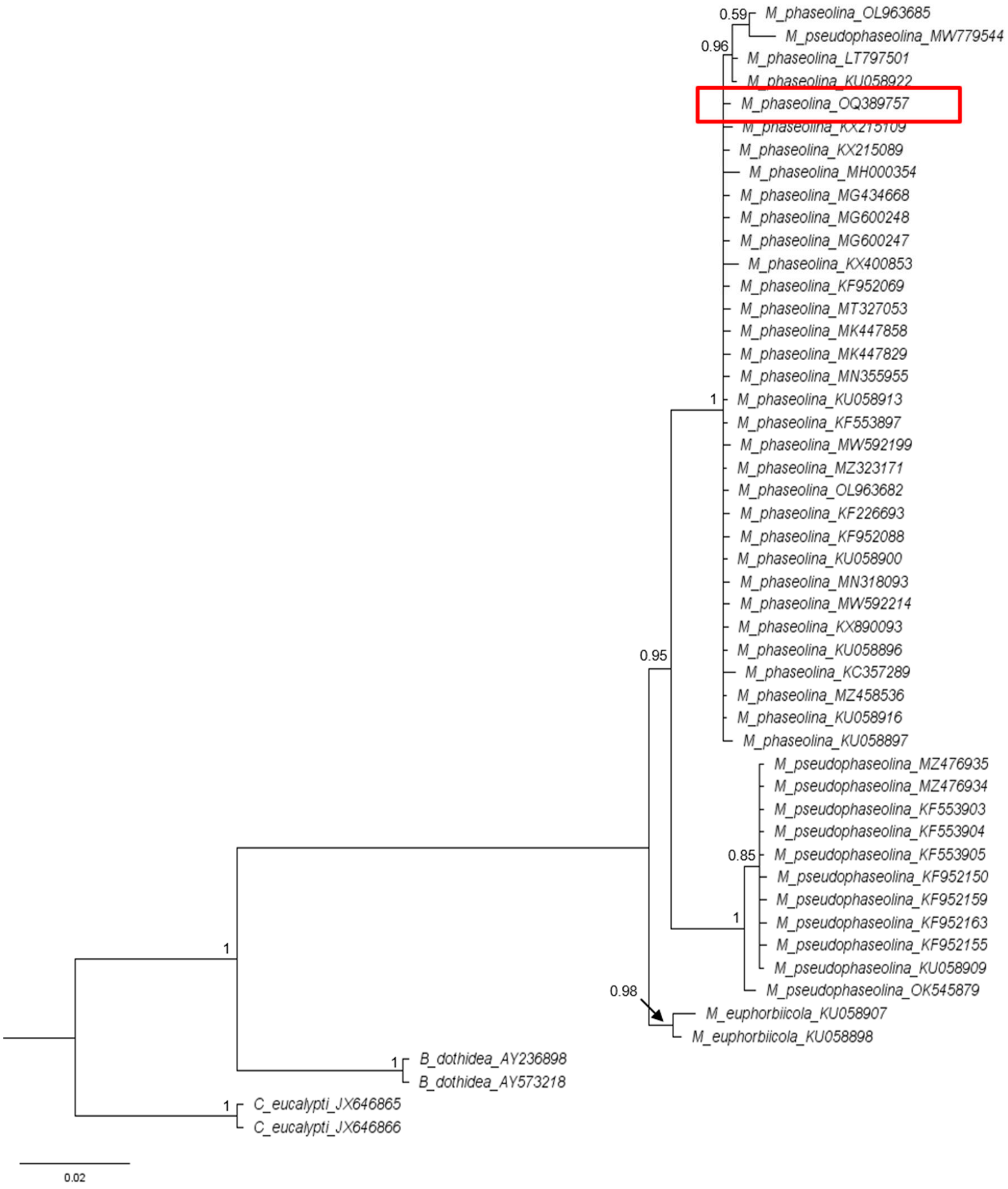

Phylogenetic placement of isolate MP1 (*Macrophomina phaseolina*) based on partial TEF1-α sequences. MP1 (GenBank OQ389757; 212 bp) clusters with *M. phaseolina* reference sequences, consistent with BLASTn identification (100% identity to MG434668). Branch support values (if available) are shown at nodes.

Supplementary Tables

Table S2. Derived lesion-length parameters by genotype and assessment time (DPI).

Genotype	Mean lesion (C)	Mean lesion (S)	ΔL	ΔL (%)
**3 DPI**				
G1	0.190	4.050	3.860	2031.6
G2	0.200	3.750	3.550	1775.0
G3	0.095	4.120	4.025	4236.8
G4	0.215	2.395	2.180	1014.0
G5	0.185	3.880	3.695	1997.3
G6	0.200	4.095	3.895	1947.5
G7	0.210	3.960	3.750	1785.7
G8	0.195	3.820	3.625	1859.0
G9	0.210	2.100	1.890	900.0
**7 DPI**				
G1	1.460	3.200	1.740	119.2
G2	1.300	2.450	1.150	88.5
G3	1.520	3.350	1.830	120.4
G4	1.410	2.980	1.570	111.3
G5	2.625	3.750	1.125	42.9
G6	1.880	4.205	2.325	123.7
G7	1.720	3.460	1.740	101.2
G8	1.600	3.150	1.550	96.9
G9	2.075	2.195	0.120	5.8
**10 DPI**				
G1	1.585	3.100	1.515	95.6
G2	1.325	2.740	1.415	106.8
G3	1.670	3.950	2.280	136.5
G4	1.480	3.200	1.720	116.2
G5	2.645	3.900	1.255	47.4
G6	1.990	4.210	2.220	111.6
G7	1.810	3.520	1.710	94.5
G8	1.690	3.100	1.410	83.4
G9	2.215	2.240	0.025	1.1
**14 DPI**				
G1	1.945	3.495	1.550	79.7
G2	2.105	2.740	0.635	30.2
G3	2.250	4.980	2.730	121.3
G4	2.180	3.300	1.120	51.4
G5	3.160	4.050	0.890	28.2
G6	2.390	4.600	2.210	92.5
G7	2.260	3.890	1.630	72.1
G8	2.200	3.450	1.250	56.8
G9	2.330	2.400	0.070	3.0
**21 DPI**				
G1	2.480	4.020	1.540	62.1
G2	2.245	2.970	0.725	32.3
G3	2.650	6.210	3.560	134.3
G4	2.580	3.600	1.020	39.5
G5	3.240	4.200	0.960	29.6
G6	2.750	5.020	2.270	82.5
G7	2.600	5.500	2.900	111.5
G8	2.540	3.950	1.410	55.5
G9	2.680	2.740	0.060	2.2

For each genotype (G1–G9) and each assessment time (3, 7, 10, 14, and 21 days post-inoculation; DPI), the table reports mean stem lesion length under control conditions (C) and after cold stress (S), the treatment-induced increase ($\Delta L = S - C$), and the relative increase ($\Delta L\% = 100 \times (S - C)/C$). Lesion length is given in **cm** (means across plants within replicate; see Methods).

Table S3. Time-course indices of lesion development by genotype.

Genotype	AULPC ©	AULPC (S)	CPI (%)
G1	33.54	52.62	56.8
G2	26.78	40.47	51.1
G3	31.92	63.11	97.7
G4	29.64	43.21	45.8
G5	38.85	57.33	47.5
G6	35.17	59.84	70.1
G7	30.96	54.47	76.0
G8	28.41	46.92	65.2
G9	27.88	29.06	7.5

The table summarizes lesion progression under control (C) and cold-stress (S) conditions using the area under the lesion progress curve (AULPC; trapezoidal integration of mean lesion length across 3–21 DPI) and the cold-predisposition index (CPI, %), defined as 100 × (AULPC_S − AULPC_C)/AULPC_C. Higher AULPC indicates faster and/or greater lesion expansion over time, while higher CPI indicates a stronger cold-stress-associated increase in disease development.

Cross-references in the manuscript

Methods → Statistics: "Definitions and calculation details for ΔL, ΔL%, AULPC, and CPI are provided in Supplementary Methods S1."

Results (first mention of relative/treatment-induced increase): "Full per-genotype calculations are provided in Tables S2–S3 (Supplementary Methods S1)."

Discussion (practical implications): "See Supplementary Methods S1 for index definitions and examples."

S4. Supplementary Table S4 — Genotype metadata (anonymized)

This table provides non-identifying metadata for each coded genotype (G1–G9) used in the study. The public version preserves anonymization to avoid brand-related bias and to comply with research-use agreements. Full unblinded identifiers (trade names/line codes/suppliers) can be provided to the Editor upon confidential request.

Code (public)	Maturity group (MG)	Origin category (AIO breeding / External supplier)	Status (Registered cultivar / Breeding line)	Country/region of origin	Seed lot year	Supplier type (certified / research-use)
G1 – (IKA)	MG 0-I	AIO breeding	Registered	Croatia	2024	Certified
G2 – (TENA)	MG 0-I	AIO breeding	Registered	Croatia	2024	Certified
G3 – (TOMA)	MG 0	AIO breeding	Registered	Croatia	2024	Certified
G4 – (SONJA)	MG 0	AIO breeding	Registered	Croatia	2024	Certified
G5 – (SUNCE)	MG 0-I	AIO breeding	Registered	Croatia	2024	Certified
G6 – (OS NEVENA)	MG 0	AIO breeding	Registered	Croatia	2024	Certified
G7 – (EMA)	MG 00–0	AIO breeding	Registered	Croatia	2024	Certified
G8 – (OS ĐURĐICA)	MG 0–I	AIO breeding	Registered	Croatia	2024	Certified
G9 – (KORANA)	MG 00	AIO breeding	Registered	Croatia	2024	Certified

Note: Public anonymization preserves scientific neutrality; see Methods for MG mapping rationale. If journal policy requires full identifiers post-acceptance, this table can be replaced with the unblinded version.
